# Advances in the mechanisms and applications of inhibitory oligodeoxynucleotides against immune-mediated inflammatory diseases

**DOI:** 10.3389/fphar.2023.1119431

**Published:** 2023-02-07

**Authors:** Hongrui Wang, Yingying Su, Duoduo Chen, Qi Li, Shuyou Shi, Xin Huang, Mingli Fang, Ming Yang

**Affiliations:** ^1^ Department of Molecular Biology, College of Basic Medical Sciences, Jilin University, Changchun, Jilin, China; ^2^ Department of Anatomy, College of Basic Medical Sciences, Jilin University, Changchun, Jilin, China

**Keywords:** inhibitory oligodeoxynucleotides, antisense oligodeoxynucleotides, immune responses, inflammatory diseases, immunosuppressive oligodeoxynucleotide, aptamer

## Abstract

Inhibitory oligodeoxynucleotides (ODNs) are short single-stranded DNA, which capable of folding into complex structures, enabling them to bind to a large variety of targets. With appropriate modifications, the inhibitory oligodeoxynucleotides exhibited many features of long half-life time, simple production, low toxicity and immunogenicity. In recent years, inhibitory oligodeoxynucleotides have received considerable attention for their potential therapeutic applications in immune-mediated inflammatory diseases (IMIDs). Inhibitory oligodeoxynucleotides could be divided into three categories according to its mechanisms and targets, including antisense ODNs (AS-ODNs), DNA aptamers and immunosuppressive ODNs (iSup ODNs). As a synthetic tool with immunomodulatory activity, it can target RNAs or proteins in a specific way, resulting in the reduction, increase or recovery of protein expression, and then regulate the state of immune activation. More importantly, inhibitory oligodeoxynucleotides have been used to treat immune-mediated inflammatory diseases, including inflammatory disorders and autoimmune diseases. Several inhibitory oligodeoxynucleotide drugs have been developed and approved on the market already. These drugs vary in their chemical structures, action mechanisms and cellular targets, but all of them could be capable of inhibiting excessive inflammatory responses. This review summarized their chemical modifications, action mechanisms and applications of the three kinds of inhibitory oligodeoxynucleotidesin the precise treatment of immune-mediated inflammatory diseases.

## 1 Introduction

Nucleic acids derived from microorganisms can trigger powerful immune responses through a variety of signaling sensors, including Toll-like receptors (TLRs), cyclic GMP-AMP Synthase (c-GAS), melanoma differentiation-associated protein 5 (MDA5), retinoid-induced gene-1 (RIG-I) and oligomerization domain (NOD)-like receptors (NLRs) (Chen et al., 2019; Natarajan and Ranganathan 2020; Tarigan et al., 2020). Unmethylated cytosine-guanine dinucleotide-containing oligodeoxynucleotide (CpG ODN), which mimics bacterial DNA binding to TLR9 and leads to activate the innate and adaptive immune response in many immune cells (Yagci et al., 2010; Scheiermann and Klinman 2014; Lai C. Y et al., 2019). In contrast, some DNA oligodeoxynucleotides (ODNs) have been evaluated for their ability to inhibit immune activation, which are called inhibitory ODNs. Generally, inhibitory ODNs are short synthetic single-stranded DNA molecules that have dozens of deoxynucleotides in length and specific sequences with negative regulation of immune signaling (Hammond et al., 2021).

In the past few decades, inhibitory ODNs have attracted great attention because their potential therapeutic applications in immune-mediated inflammatory diseases (IMIDs) (Lai C. Y et al., 2019; Wan et al., 2021). A bunch of preclinical studies suggest that inhibitory ODNs could prevent IMIDs, including inflammatory disorders and autoimmune diseases (Crooke et al., 2017). For example, a sequence with repetitive TTAGGG motifs derived from mammalian telomere DNA significantly downregulated the production of pro-inflammatory cytokines induced by CpG ODNs or cellular damage-related molecular patterns (DAMPs) released from injury cells (Gursel et al., 2003). Moreover, some ODN drugs have been approved for marketing. For instance, Fomivirsen, an antisense oligodeoxynucleotide (AS-ODN) was developed for the treatment of patients with cytomegalovirus (CMV) retinitis, which became the first ODN drug to be approved by the Food and Drug Administration (FDA) in 1998 (Roehr 1998). With the innovation of genomics and various new nucleotide synthesis technologies, synthetic inhibitory ODNs have shown rapid development in the application of translational and precision medicine since their regulation in specific cellular processes (Hammond et al., 2021). In this review, we define three classifications of inhibitory ODNs based on their mechanisms of action and targets, and then discuss the relationship of chemical modified characteristics with the function of ODNs. Furthermore, we summarized their potential therapeutic applications against IMIDs as well.

## 2 Main classifications of inhibitory ODNs

### 2.1 Antisense oligodeoxynucleotides

Antisense oligodeoxynucleotides (AS-ODNs) are short, synthetic, single-stranded oligodeoxynucleotides that are complementary to specific sequences of target RNA (Gheibi-Hayat and Jamialahmadi 2021). They alter the target RNA and protein expression by interfering with RNA transport, splicing or translation (Kim et al., 2019). Therefore, AS-ODNs have been evaluated as candidate therapeutic agents to silence target genes in viral infections, autoimmune disease, cancer and genetic disorder related diseases due to their high degree of selectivity and low toxicities (Bennett 2019). Moreover, it was also shown to be a powerful tool for gene function analysis in the medical sciences (Dzialo et al., 2017).

### 2.2 DNA aptamers

DNA aptamers are oligodeoxynucleotide compounds synthesized from single-stranded DNA molecules, usually consisting of 15–100 nucleotides, with the ability to form secondary and tertiary structures that bind specifically to target molecules (Keijzer et al., 2021; Chen et al., 2022). Aptamers are a new class of ligands that can bind to targets in the range of picomoles and are often compared with antibodies (Morita et al., 2018). Both aptamers and antibodies are biomolecules that bind targets with high affinity and can be used to modulate target functions for diagnosis and treatment of diseases. Compared to antibodies, DNA aptamers exhibit significant advantages including small size, mature and rapid screening process, easy synthesis and chemical modification, as well as low immunogenicity (Table 1). These advantages make aptamers as a promising alternative to antibodies (Dehghani et al., 2018; Ahmadyousefi et al., 2019). DNA aptamers can be specifically used to bind with corresponding proteins, peptides, small molecules and other targets, inhibiting their biological functions, affecting their activity, and achieving the purpose of diseases diagnosis and treatment (Chen et al., 2017).

**TABLE 1:** Main advantages of aptamers compared to antibodies.

Features	Aptamers	Antibodies
Size	Small	Large
Stability	Temperature resistant	Temperature sensitive and easily denatured
Chemical modification	Modifications can enhance structural and functional properties	Modifications often lead to reduced activity
Storage condition	Normal temperature preservation	Frozen preservation
Synthesis cycle	2–8 weeks	More than 6 months
Economic cost	Cheap	Laborious and expensive
Batch variance	No	Yes

### 2.3 Immunosuppressive oligodeoxynucleotides

Immunosuppressive oligodeoxynucleotide (iSup ODN) refers to a class of artificially synthesized single-stranded DNA molecules with a length of tens of nucleotide sequence to function the negative regulation of immune response (Yamada et al., 2004). Compared to CpG ODNs derived from exogenous microorganisms, iSup ODN sequences are mostly derived from mammal genomes. For example, the DNA fragments from calf-thymus and human placenta were shown to inhibit bacterial DNA-induced production of interleukin-12 (IL-12) in murine macrophages (Pisetsky 2000). Mammalian telomere derived TTAGGG repeats were found to inhibit CpG ODN induced production of interleukin-6 (IL-6), IL-12, and interferon-α (IFN-α) in mouse spleen cells (Gursel et al., 2003). Similarly, human telomere derived ACCCCTCT repeats were also found to inhibit CpG ODN induced proliferation of human peripheral blood monocytes and type I IFN production (Yang and Yang 2010). These findings suggest that some iSup ODNs may have some self-regulatory mechanism, which can inhibit over-activated immune response in the body by means of protective negative feedback regulation. Thereby, the iSup ODNs have potential applications in the treatment of immune-mediated inflammatory diseases, but many biological functions of iSup ODNs remain to be developed. Further exploration and discussion of iSup ODN can lay a theoretical foundation for the development of a new and effective iSup ODN drug for treating immune-related diseases in the future.

## 3 Chemical modifications of inhibitory ODNs

Single-stranded ODNs face several challenges that complicate drug development. The unfavorable properties of ODNs include: 1) degradation by nucleases when introduced into biological systems, 2) poor uptake through cell membranes, 3) unfavorable biodistribution and pharmacokinetic properties and 4) suboptimal binding affinity for complementary sequences (Crooke et al., 2017; Shi et al., 2021). Thereby, inhibitory ODNs are usually chemically modified in several ways to ensure them with properties such as increased the resistance to nucleases and improved the target binding affinity (Honcharenko et al., 2022). Different modifications confer the ODNs with diverse properties, and modify the ODNs in ways that complicate their synthesis or interfere with the mechanisms by which they exert their effect (Table 2).

**TABLE 2:** Chemical modifications of inhibitory oligonucleotides.

Name	Mechanism	Properties	References
Phosphorothioate (PS)	RNase H1 cleavage	Enzymatic stability	Shen and Corey (2018)
2′-O-methyl (2′-O-Me)	Steric hindrance/splice modulation	Higher binding affinity, enzymatic stability, reduced immune stimulation	Odeh et al. (2019)
2′-O-methoxyethyl (2′-O-MOE)	Steric hindrance/splice modulation	Higher binding affinity, enzymatic stability, reduced immune stimulation	
Locked nucleic acid (LNA)	Steric hindrance/RNase H1 cleavage	Higher binding affinity, enzymatic stability	Quemener et al. (2022)
Peptide nucleic acid (PNA)	Steric hindrance/splice modulation	Enzymatic stability, higher binding affinity, no immune activation	
Phosphorodiamidate morpholino oligonucleotides (PMO)	Steric hindrance/splice modulation	Improved aqueous solubility, higher binding affinity	

First-generation chemistries include the widely used phosphate backbone modifications, e.g., phosphorothioate (PS). PS modification, replacement of a non-bridging phosphodiester oxygen by sulfur, is the most widely used single alteration in nucleic acid drug development (Eckstein 2014). PS linkages serve two purposes. First, PS modification could increase the ODN stability toward digestion to nucleases. The modification transforms DNA sequences with half-lives of minutes to half-lives of days. Second, it can increase the binding to proteins, especially serum proteins. Increased binding to serum proteins preserves ODNs in circulation, and slows removal by the liver and kidney to prolong the time available for uptake into target tissues (Dowdy 2017). However, this modification often reduces the affinity to the targets. By contrast, DNA modifications with second-generation chemistries exhibit the increase of binding affinity to RNA and further improve the nuclease resistance (Kajino and Ueno 2021). Generally, the main second-generation chemistries include ribose modifications at the 2ʹ-position of RNA and 2ʹ-position of DNA, of which the 2ʹ-O-methyl (2ʹ-O-Me) and 2ʹ-O-methoxyethyl (2ʹ-O-MOE) modifications are the most commonly used types (Quemener et al., 2022).

In addition, other types of chemical modifications have been developed, such AS locked nucleic acid (LNA), peptide nucleic acid (PNA), and phosphorodiamidate morpholino oligonucleotides (PMO) (Hagedorn et al., 2018). PNA can regulate gene expression or induce mutations by invading chromosomal double-stranded DNA. LNA can freely bind to DNA to form chimeric interspace molecules, promoting affinity and nuclease resistance to Ribonuclease H (RNase H) (Urban and Noe 2003). The novel chemical modification can resist the degradation of nuclease and peptidase, improving the nuclease stability, target affinity and pharmacokinetic characteristics of inhibitory ODNs (Araie et al., 2021). Chemical modifications carried out over the past decades have given inhibitory ODNs greater specificity and the ability to function more stably, reliably and safely (Fang et al., 2020).

## 4 Action mechanisms and cellular endocytosis of inhibitory ODNs

AS-ODNs prevent protein translation of certain mRNA strands by binding to them, in a process called hybridization. By contrast, DNA aptamers can directly bind to specific target molecules especially proteins with high affinity and specificity. Unlike the above mentioned inhibitory ODNs, iSup ODNs play their role mainly by competitively binding receptors or inhibiting signal transduction of important factors in inflammatory pathways. Relatively, the mechanism of AS-ODN is clearer due to its widely used in basic research and clinical application.

### 4.1 Target cleavage mechanisms of AS-ODNs

#### 4.1.1 Activation of ribonuclease

**FIGURE 1:**
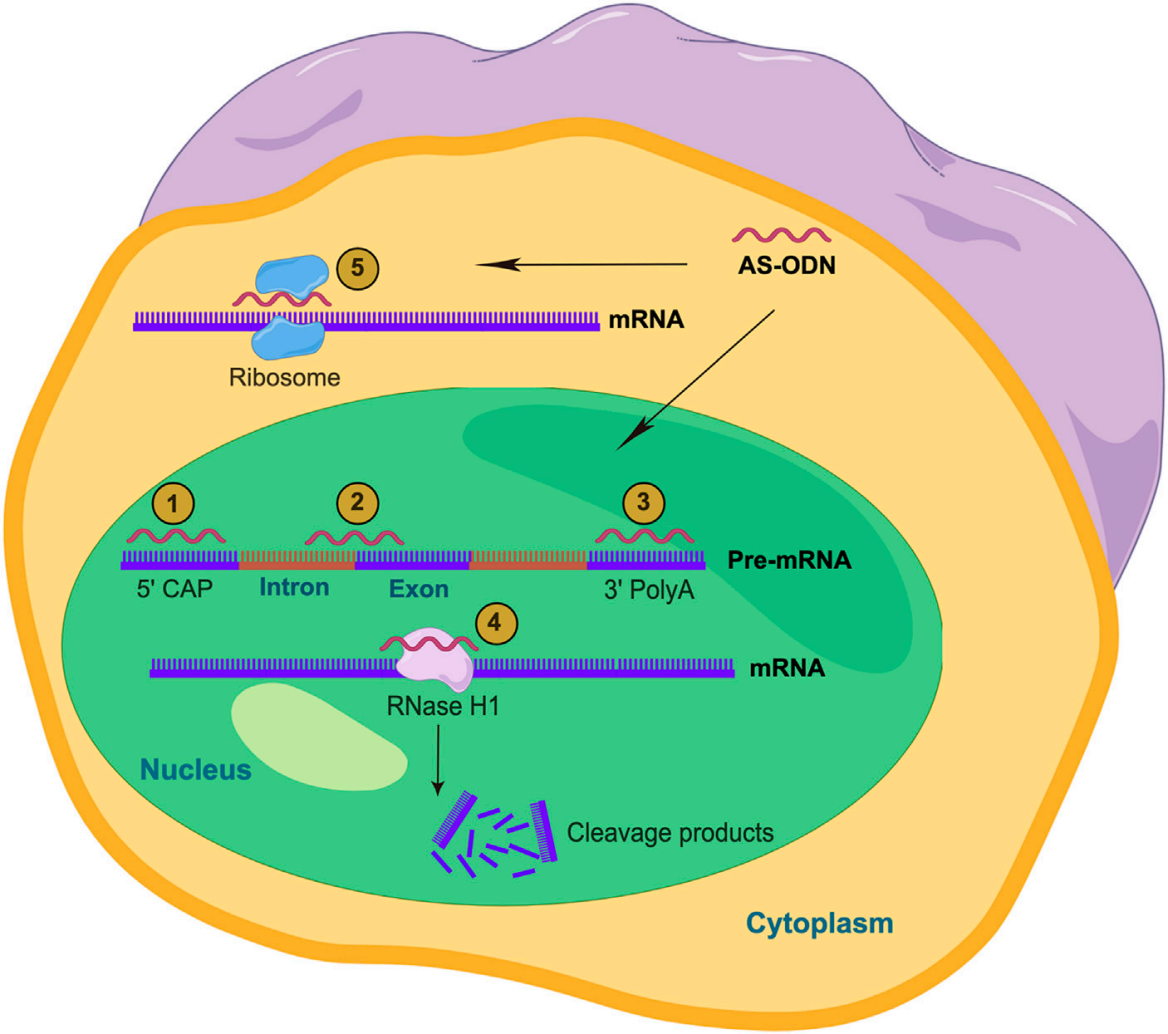
Action mechanism of AS-ODNs (By Figdraw). AS-ODNs can be designed to prevent the 5′-mRNA cap formation (1), bind to intron/exon junctions to modulate splicing processes (2), or bind to 3′ UTR to modulate polyadenylation (3). Also, AS-ODNs can be designed to activate RNase H1 and induces the cleavage of the mRNA (4). Moreover, the direct skipping of the mRNA by AS-ODNs inhibit the physical assembly of the ribosome subunits on the mRNA sequence (5).

RNase H is responsible for the degradation of RNA-DNA hybrids synthesized along the chain in the nucleus. RNase H is also present in the cytoplasm and degrades mature mRNA (Vickers and Crooke 2015). The DNA-RNA hybrid formed by the combination of the partial DNA sequence from AS-ODN and the target mRNA will attract RNase H to the site to cut and degrade the target mRNA, thereby reducing the expression of the target gene product (Lennox and Behlke 2016). Some results also showed that the important role of AS-ODN is related to the cleavage of target mRNA by RNase H in the cytoplasm, but this role is more efficient in the nucleus (Kielpinski et al., 2019) (Figure 1). In addition, some scholars believed that AS-ODN “Gapmer” contains chemically modified RNA bases located on both sides of the central 8–10 base DNA “Gap” (Monia et al., 1993). RNA bases can enhance affinity with complementary sequences and DNA bases can act as substrates of RNase H. RNase H enhances the effect of AS-ODNs by inducing cleavage of target mRNA, which is an important feature of therapeutic oligodeoxynucleotides that inhibit translation (Smolka et al., 2021).

#### 4.1.2 Splice correction for pre-mRNA

AS-ODNs can block the binding of some functional groups to the functional sequences involved in gene splicing in pre-mRNA, regulate or change the gene-splicing mode of the pre-mRNA, and induce the production of new functional protein heteroform (Blum et al., 2015) (Figure 1). During exon hopping therapy, AS-ODNs bind to the pre-mRNA transcript to correct the damaged reading frame and produce a brief but functional protein (Havens et al., 2013). In exon retention therapy, AS-ODNs bind to the pre-mRNA site and prevents the spliceosome and splice factors from accessing the transcription site (Bauman et al., 2009).

#### 4.1.3 Formation of the DNA triple helix structure

Triple helix-forming oligonucleotides could bind to the major groove of double-stranded purine-rich chain to form Triple-stranded DNA through Hoogsteen or reverse Hoogsteen hydrogen bonds (Casey and Glazer 2001). Thus, they can be used in anti-gene strategies as an alternative to antisense technology. Similarly, AS-ODNs can be inserted into the structural groove of the DNA double helix, leading to the formation of the triple helix structure in the nucleus, especially in the high-value region of purine-pyrimidine pairing, resulting in cleavage of the target DNA and thus impeding the transcription process (Ricciardi et al., 2014) (Figure 1).

### 4.2 Cellular endocytosis of DNA aptamers

**FIGURE 2:**
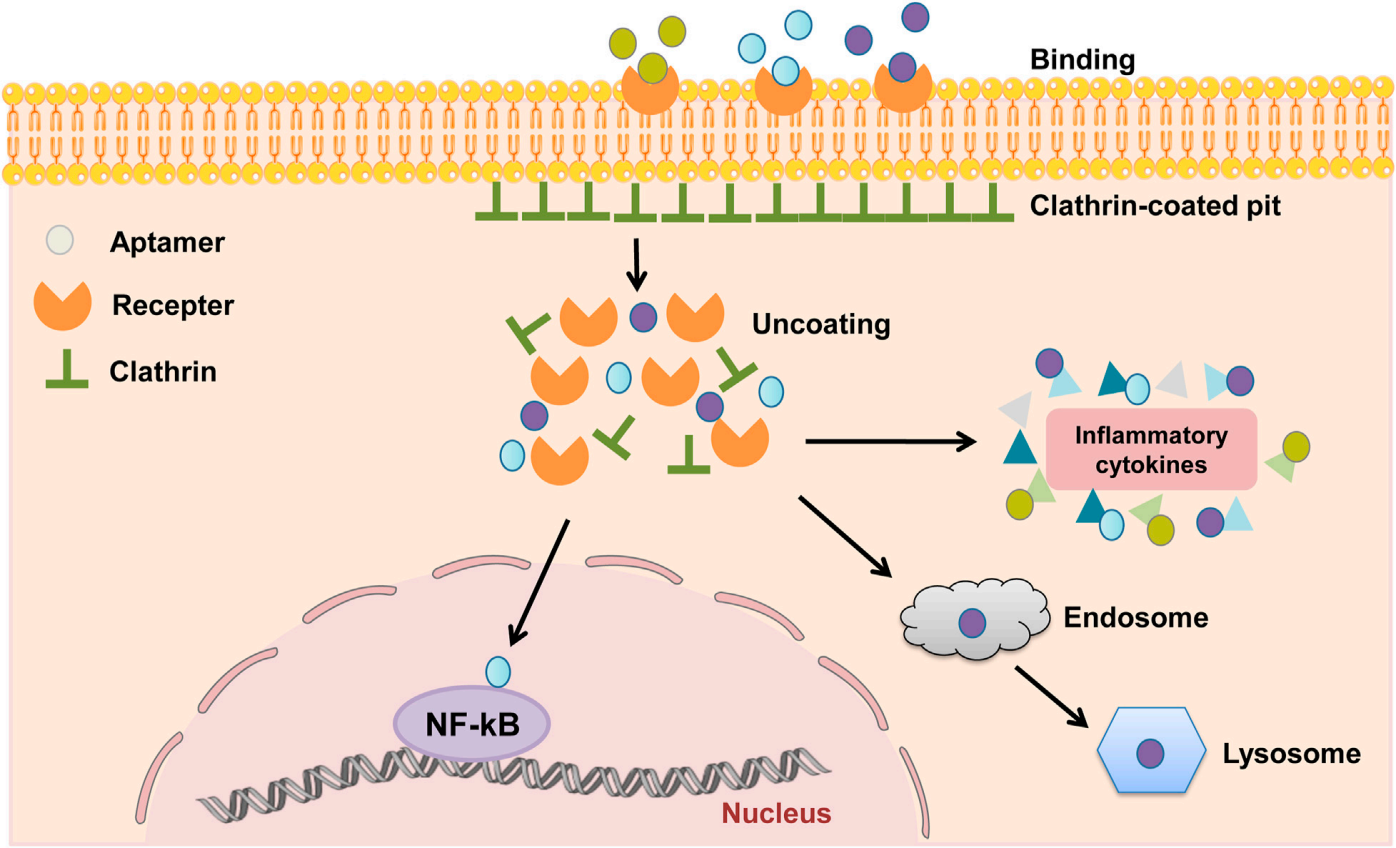
Mechanisms of clathrin-dependent endocytosis of aptamers. The binding of the aptamers and receptors initiate the formation of clathrin-coated pit, followed by clathrin-coated vesicle budding. Once detached from the membrane, the clathrin coat is disassembled. The aptamers can bind to the proteins in the nuclear inflammatory signaling pathway, such as the NF-κB signaling pathway, and can also bind to inflammatory cytokines such as TNF-α, IFN-γ, and IL-6 in the cytoplasm to play an inhibitory role. Meanwhile, some aptamers are degraded due to clathrin-dependent endocytosis and eventual fusion with endosomes and lysosomes.

DNA aptamers are developed for cell surface receptors that bind to protein target sites through three-dimensional electrostatic interactions and bag-like structures for targeted delivery. Therefore, cellular internalization of DNA aptamers is an important way to develop targeted drug delivery systems *in vivo* (Wan et al., 2019). Studies have shown that endocytosis is the main pathway of cell internalization of different aptamers, including phagocytosis, pinocytosis, clathrin-mediated endocytosis (CME) and alveolar protein-mediated endocytosis (Kaksonen and Roux 2018; Wan et al., 2019). The process by which aptamers are taken into cells depends on their targets but is typically clathrin-mediated endocytosis or macropinocytosis. The clathrin-mediated endocytosis depends on actin and dynamin function, whereas macropinocytosis does not (Li et al., 2017; Wan et al., 2022). Depending on the target or cell type, the main internalization mechanism of aptamers is clathrin-dependent (Figure 2). The binding of the aptamer and receptor initiate formation of the clathrin-coated pit, followed by clathrin-coated vesicle budding. Once detached from the membrane, the clathrin coat is disassembled. Clathrin-dependent endocytosis ends in fusion with endosomes and lysosomes. Typical adaptations for demonstrated clathrin-dependent internalization are Burkett lymphoma cell-specific DNA aptamer and anti-protein tyrosine kinase 7 aptamer (Opazo et al., 2015; Wang et al., 2016). So far, there are relatively few studies on the internalization mechanism of aptamers. Therefore, strategies can be adopted to further explore the relevant mechanisms, screen suitable ligands and use them as targeted delivery tools for corresponding cells.

### 4.3 Immunosuppressive mechanisms of iSup ODNs

**FIGURE 3:**
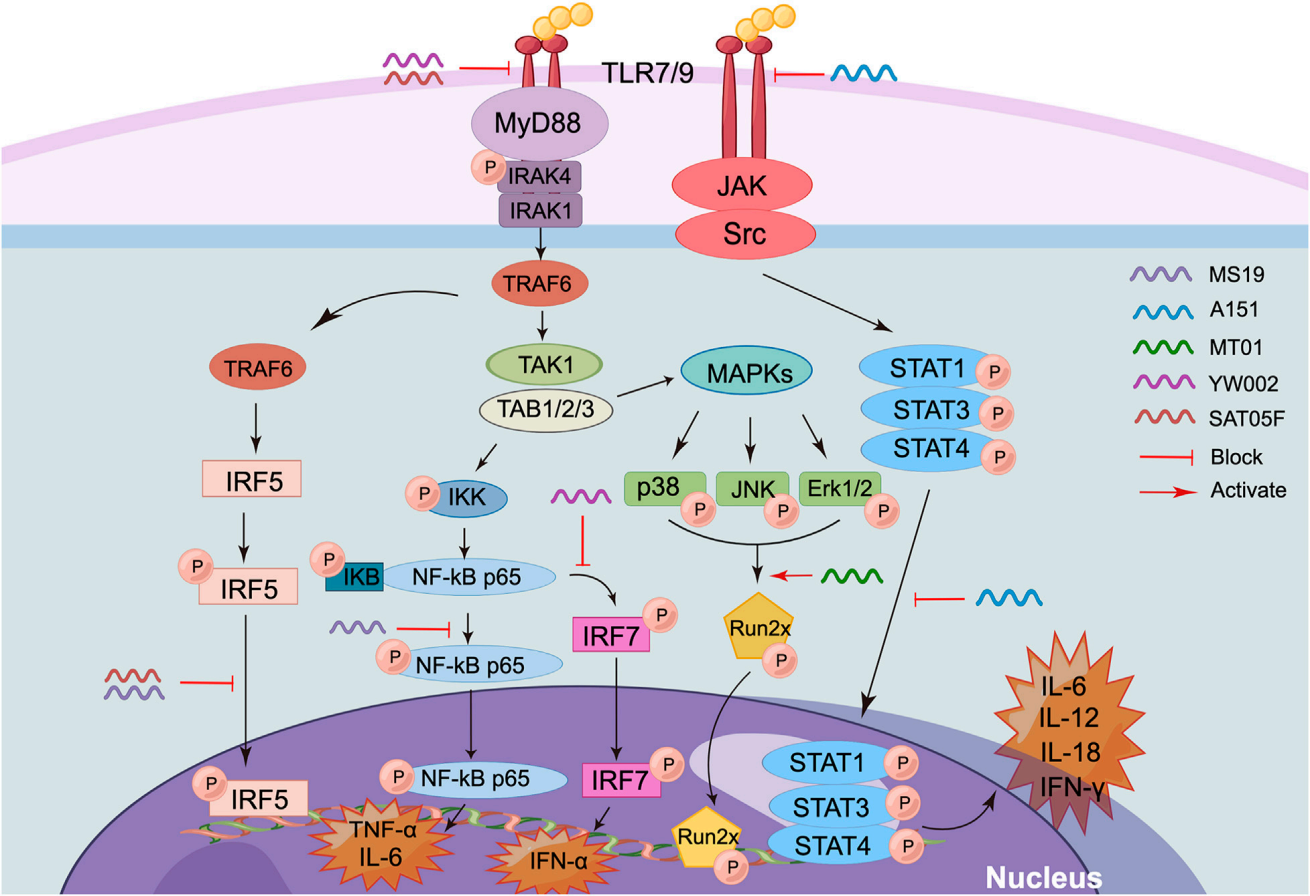
Action mechanism of iSup-ODNs (By Figdraw). (1) MS19 inhibits the expression and nuclear translocation of IRF5 and phosphorylation of p65 NF-κB, thus extensively inhibiting the expression of inflammatory cytokines IL-6 and TNF-α. (2) A151 inhibits the activation of TLR7/9, inhibits the phosphorylation of STAT1, STAT3, and STAT4, and downregulates the expression of inflammatory cytokines such as IFN-γ, IL-12, IL-6, and IL-18. (3) MT01 activates Runx2 phosphorylation through MAPKs signaling pathway, delays the aging rate of DPSCs, and then inhibits the development of pulpitis. (4) YW002 can induce the downregulation of TLR9 and TNF-α. (5) SAT05f can downregulate IFN-α production by inhibiting TLR7/9 signaling pathway.

iSup ODNs can be divided into two categories based on sequence and functional characteristics (Figure 3) (Fehér 2019). The first type is the guanine (G)-rich ODNs. Usually, G-rich iSup ODN can inhibit the activation of TLR9, especially inhibit the immune responses induced by CpG ODNs, such as B cell proliferation, the production of IL-6, IL-12, IFN-γ, and IFN-α (Stunz et al., 2002; Barrat et al., 2005). Moreover, the increasing of poly-G enhances its immunosuppressive activity (Stunz et al., 2002). As a typical inhibitory ODN with poly G, A151 with tandem repeat sequence “TTAGGG” derived from the telomeres of mammalian chromosomes was well investigated (Khan et al., 2022). It has been reported that A151-pretreated dendritic cells and macrophages have reduced their ability to produce type I IFN and tumor necrosis factor α (TNF-α) when activated by cytoplasmic dsDNA. Moreover, A151 blocked IFN-γ, IL-12, IL-6, and other signaling pathways by inhibiting the phosphorylation of signal transducer and activator of transcription 1 (STAT1), STAT3 and STAT4, thereby downregulating immune activation (Peter et al., 2008). To clarify the mechanism of iSup ODNs blocking STAT phosphorylation, it was found that A151 was highly specific for intracellular binding to STAT1, STAT3, and STAT4, but did not bind to nuclear factor kappa B (NF-κB) or other molecules in the signaling cascade. Also, A151 inhibited macrophage and dendritic cells from producing mature IL-1β and IL-18 by binding to the AIM2 inflammasome in competition with the irritating ODN (Kaminski et al., 2013). Notably, A151 ODN requires G-tetramer formation to maintain its broad immunosuppressive activity (Klinman et al., 2003).

Some non-G-rich ODN also can inhibit the activation of immune response. For example, SAT05f was designed by our laboratory, which can selectively inhibit the activation of TLR7/9 signaling pathway, subsequently downregulate the production of IFN-α production. Moreover, the inhibition of TLR9 activation is closely related with the blockade of endoplasmic reticulum transmembrane protein Unc93B1 intracellular transport induced by SAT05f (Zhang et al., 2014). MT01, another iSup ODN designed in our lab, can inhibit the proliferation of human peripheral blood mononuclear cells (PBMCs) induced by CpG ODN, and can inhibit the production of type I IFN and B cell activation induced by TLR agonists (Zheng et al., 2020). Similarly, it was showed that MT01 activated Runx2 phosphorylation through MAPKs signaling pathway, delayed the aging rate of dental pulp stem cells (DPSCs), and inhibited the development of pulpitis (Shen et al., 2012). However, the mechanism of iSup ODN is not yet fully understood and needs to be further explored and clarified by researchers.

## 5 Therapeutic applications of inhibitory ODNs in immune-mediated inflammatory diseases

Inflammation should be precisely controlled in quantitative, qualitative and temporal terms, improper control could compound the disease processes or cause severe IMIDs (Salazar et al., 2017; Tanaka et al., 2018; Jang et al., 2021). In fact, immune-mediated inflammatory diseases comprise a common, clinically diverse group of conditions for which there are no current cures. In the past few decades, the use of synthetic ODNs has made breakthroughs, providing new support for the treatment of IMIDs by improving the chemical modifications in these molecules to make them more stable and specific. Several AS-ODNs and DNA aptamers have entered different stages of clinical trials, and some have been approved by the FDA (Table 3). Likewise, the immunosuppressive activity of many iSup ODNs has been fully demonstrated in preclinical studies (Table 3).

**TABLE 3:** Current synthetic inhibitory oligonucleotides in clinical and preclinical trials.

ODN type	Drug name	Disease	Status	Reference
AS-ODN	Vitravene	CMV retinitis	FDA approved	Roehr (1998)
AS-ODN	Inotersen	Hereditary transtherthyretin-mediated amyloidosis	FDA approved	Keam (2018)
AS-ODN	ISIS 2922	CMV-induced retinitis in AIDS	Phase III	Cinatl et al. (2000)
AS-ODN	ISIS 2302	Ulcerative colitis, Rheumatoid arthritis	Phase III	Scarozza et al. (2019)
AS-ODN	ISIS 104838	Rheumatoid arthritis	Phase III	Narayanan et al. (2018)
AS-ODN	EPI-2010	Asthma	Phase II	Ball et al. (2004)
AS-ODN	TPI ASM8	Allergic inflammation	Phase II	Gauvreau et al., 2011; Imaoka et al., 2011
AS-ODN	Gem92	HIV	Phase I	Zheng (1999)
AS-ODN	AR 177	HIV	Phase I	Smart (1996)
AS-ODN	LNA-AS-ODN	Osteoarthritis	Pre-clinical	Nakamura et al. (2019)
Aptamer	Pegaptanib	Neovascular AMD	FDA approved	Chapman and Beckey (2006)
Aptamer	Macugen	Age-Related Macular Degeneration	FDA approved	Vavvas and D'Amico (2006)
Aptamer	ARC1905	Age-Related Macular Degeneration	Phase I	Leung and Landa (2013)
Aptamer	Fovista	Age-Related Macular Degeneration	Phase III	Tolentino et al. (2015)
Aptamer	Zimura	Geographic Atrophy Macular Degeneration	Phase II	Jaffe et al. (2021)
Aptamer	AS1411	Acute Myeloid Leukemia	Phase II	Yazdian-Robati et al. (2020)
Aptamer	NOX-E36	Chronic Inflammatory Diseases	Phase I	Baeck et al. (2012)
Aptamer	GP-120	HIV	Pre-clinical	Wang et al. (2019)
Aptamer	TLR4	brain damage after cerebral hemorrhage	Pre-clinical	Fernández et al. (2018)
Aptamer	IL-23	brain inflammation	Pre-clinical	Shahdadi Sardou et al. (2020)
Aptamer	RA10-6	Synovial inflammation in mice with osteoarthritis	Pre-clinical	Chen et al. (2011)
Aptamer	Apt-TNF-α	ALI, ALF	Pre-clinical	Lai W. Y et al. (2019)
iSup-ODN	STA05F	SLE, SIRS	Pre-clinical	Dong et al. (2005); Yazar et al. (2020)
iSup-ODN	A151	SLE, Atherosclerosis	Pre-clinical	Cheng et al. (2008)
iSup-ODN	ODN1411	RA	Pre-clinical	Sacre et al. (2016)
iSup-ODN	MT01	Periodontitis	Pre-clinical	Shen et al. (2012)
iSup-ODN	MS19	Sepsis	Pre-clinical	Gao et al. (2017)
iSup-ODN	rODN M1	IAV-induced acute ALI	Pre-clinical	Meng et al. (2017)
iSup-ODN	YW002	Alcoholic hepatitis	Pre-clinical	Wang et al. (2012)
iSup-ODN	ODN IRS954	SLE	Pre-clinical	Barrat et al. (2005)

### 5.1 AS-ODNs

AS-ODN is of great significance for the treatment of inflammatory diseases, and its mediated intervention has become an important therapeutic method for targeting gene expression operations (Zamecnik and Stephenson 1978; Keating 2014; Crooke et al., 2017). Some recent studies have displayed LNA modified AS-ODNs have considerable therapeutic effects for spinal cord injury, pulmonary fibrosis (Liu et al., 2010) and osteoarthritis (Nakamura et al., 2019). Some AS-ODNs also have therapeutic effects in the field of inflammatory bowel disease (IBD). As we known, IBD is a chronic, recurrent, inflammatory gastrointestinal disease (Seyedian et al., 2019). ISIS 2302 (Alicaforsen) is an RNase H-dependent 20-base-long antisense thiophosphoric oligonucleotide that inhibits human intercellular adhesion molecule-1 (ICAM-1), and it also was the first AS-ODN to be used for the treatment of IBD (Glover et al., 1997). Moreover, a specific AS-ODN targeting Smad7 has been reported to restore smad2/3 phosphorylation, leading to a reduction in inflammatory cytokines in IBD mucosal cells (Monteleone et al., 2001).

In addition, AS-ODN directly acts on viral genomic RNA or transcripts and can be rationally designed for the treatment of human immunodeficiency virus (HIV), hepatitis B and C viruses (HBV and HCV), herpes viruses or any new virus (Panda et al., 2021). Fomivirsen (Vitravene), an anti-cytomegalovirus (CMV) agent, is the first AS-ODN to be approved by the FDA as an antiviral therapy. Fomivirsen is an oligonucleotide complementary to the major immediate early region 2 (IE2) of CMV mRNA. In the treatment of CMV retinitis in immunodeficient HIV patients, Fomivirsen can be used to help patients who are resistant to other treatments for CMV (Stein and Castanotto 2017). Thus, AS-ODN can be used as an antiviral agent against acute and chronic inflammation caused by virus infections.

Allergic asthma is also as typical IMIDs. Adenosine A1 receptor has been proposed as a therapeutic intervention target for asthma (Haskó et al., 2008; Bhalla et al., 2020). EPI-2010 is a 21-mer PS-modified respiratory AS-ODN developed by Epigenesis Pharmaceuticals, which reduces airway inflammation and bronchoconstriction by selectively reducing the expression of adenosine A1 receptors *in vivo*, and increases the level of surfactant in experimental models of allergic asthma (Ball et al., 2004). As we known, granulocyte-macrophage colony-stimulating factor (GM-CSF), IL-3, and IL-5 play a key role in allergic inflammation. They mediate their effect *via* receptors with a common beta subunit (betac) that transduces cell signaling. TPI ASM8 is an AS-ODN targeting betac with PS modification. It has been confirmed that TPI ASM8 downregulated the biologic activities of GM-CSF, IL-3, and IL-5 simultaneously by inhibiting betac mRNA expression with antisense technology. Moreover, TPI ASM8 has been shown to be safe and well tolerated in human trials (Gauvreau et al., 2011; Imaoka et al., 2011). Although EPI-2010 and TPI ASM8 have not been associated with any serious side effects, these AS-ODN safety studies need to last longer because asthma, COPD, and fibrosis are chronic conditions requiring long-term medication (Liao et al., 2017).

Importantly, the continued enrichment of human transcriptomic and proteomic data could facilitate the identification of promising nucleic acid targets for AS-ODNs. With the validation of new targets, and the optimization of nucleic acid-based drug delivery and modification strategies, AS-ODN has the potential to be a promising therapeutic strategy for the treatment of inflammatory diseases in clinical application.

### 5.2 DNA aptamers

DNA aptamers can change the distribution of intracellular substances and inhibit biological functions by specifically binding to corresponding proteins, peptides and small molecules, which indicates their potential in the treatment of diseases (Dantsu et al., 2021). DNA aptamers, especially those with targeting inflammatory cytokines, have a therapeutic effect on “hyperactive” immune diseases at the onset of the disease (Zhu and Chen 2018). For example, IL-1α is an essential cytokine that contributes to inflammatory responses, a naphthyl-modified DNA aptamer specifically targeting IL-1α was developed to inhibit the inflammatory signaling pathway (Ren et al., 2017). Similarly, TNF-α is also one of the most important inflammatory cytokines. The developed aptamer targeting TNF-α (Apt-TNF-α) could eliminate acute lipopolysaccharide (LPS)-induced acute lung injury (ALI) and associated acute liver failure (ALF) in mice (Lai W. Y et al., 2019). IL-17A is a pro-inflammatory factor produced by Th 17 cells, which acts as a chemical inducer that recruits immune cells, including monocytes and neutrophils, to inflammatory sites. It is reported that DNA aptamer RA10-6, which binds to IL-17A receptor, can inhibit IL-17A binding and reduce synovial inflammation in mice with osteoarthritis (Chen et al., 2011). Meanwhile, studies have shown that DNA aptamer IL-23 can detect and control brain inflammation (Shahdadi Sardou et al., 2020). Ceria nanoparticle gelatin hydrogel coated with aptamers targeting IL-17 can significantly reduce the level of inflammation in brain tissue by reducing the expression and serum concentration of IL-17, IL-10 and IL-6 (Hekmatimoghaddam et al., 2019).

Various receptors or angiogenesis are also involved in controlling the process of inflammatory diseases. Activation of the TLR4 pathways may cause inflammation, infection, and chronic disease. Thus, the development of TLR4-specific DNA aptamers has the potential for its neutralization as a therapeutic intervention (Shirey et al., 2021). Moreover, the TLR4 aptamers were highly effective in alleviating the brain damage after cerebral hemorrhage (Fernández et al., 2018). Presence of CD4 receptor on target cells is critical for productive HIV infection (Parrish et al., 2012; Zhang et al., 2021). Blocking CD4 using synthetic CD4 aptamer showed comparable cell-binding specificity as standard CD4 antibody, resulting in inhibition of viral entry and subsequent inflammatory response (Zhang et al., 2010; Fellows et al., 2020). Angiogenesis is a widely observed process in the progression of rheumatoid arthritis, IBD and neovascular age-related macular degeneration (AMD) (Aguilar-Cazares et al., 2019; Jeong et al., 2021). The increased expression of vascular endothelial growth factor A (VEGF-A) in the eye choroid promotes the formation of neovascularization by binding to its homologous receptor VEGFR2 (Yang et al., 2016). Pegaptanib is a selective anti-VEGFA aptamer that acts in the extracellular space to inhibit the 165 isoform (VEGF165), was approved by the FDA in 2004 for the treatment of all types of neovascular AMD (Xie et al., 2019). With the development of aptamer screening technology, it is believed that more and more new nucleic acid aptamer drugs will be developed and eventually used to treat diseases.

### 5.3 iSup ODNs

iSup ODN has been used in a bunch of experimental studies on the treatment of IMIDs. In our previous study, an AAAG-rich ODN designed based on the sequence of human microsatellite DNA, named as MS19, could inhibit the production of inflammatory factors in the lungs of mice caused by influenza virus or LPS, and exhibiting its therapeutic role on acute lung injury (ALI) (Gao et al., 2017; Zhang et al., 2022). Interestingly, MS19 significantly reduced the expression of inducible nitric oxide synthase (iNOS) and inflammatory cytokines not only *via* inhibiting the nuclear translocation of interferon regulatory factor 5 (IRF5), but also associated with NF-κB signaling (Gao et al., 2017; Zhang et al., 2022). In addition, MS19 was proved to alleviate the myocarditis induced by coxsackievirus B3 (CVB3) infection in mice (Nie et al., 2019). A close relationship was existed between the virus-induced ALI and the level/activity of IRF7 in local infectious sites. Thus, IRF7-rODN M1 was designed to alleviate influenza virus-induced ALI, and led to decreased mRNA levels of IFN-α, reduced neutrophil infiltration in the lungs and prolonged survival of mice (Yang et al., 2019). Another iSup ODN MT01, based on the sequence of human mitochondrial DNA, could inhibit the proliferation of PBMCs and production of type I IFN induced by influenza virus, CpG DNA and herpes simplex virus (HSV) (Yang et al., 2010). Meanwhile, MT01 could also induce differentiation of bone marrow mesenchymal stem cells (BMSCs) to osteoblasts and inhibit the alveolar bone absorption in rats with periodontitis (Shen et al., 2012). In addition, other iSup ODNs also have been screened to treat inflammatory diseases. ODN SAT05f is an oligonucleotide with CCT repeats derived from human microsatellite. It was suggested that SAT05f can recruit surface TLR9^+^ (sTLR9) neutrophils to play a protective role in the development of systemic inflammatory response syndrome (SIRS) in locally inflammatory areas (Meng et al., 2017). YW002 could alleviate the pathological changes of alcoholic hepatitis by downregulating the inflammatory factors TNF-α, IL-1β, and IL-6 (Wang et al., 2012).

In addition to above inflammatory diseases, autoimmune disease is another type of IMIDs. Systemic lupus erythematosus (SLE) is an autoimmune disease with glomerulonephritis and multifocal terminal organ damage caused by many pathogenic autoantibodies and immune complexes (Mortezagholi et al., 2017). The mammalian telomere-derived A151 can significantly reduce glomerular and tubular injury, basement membrane proliferative changes, monocyte infiltration, IgG deposition and vascular lesions in SLE model mice. Also, A151 can delay the production of proteinuria caused by glomerulonephritis, reduce the grade of proteinuria and improve the survival rate of mice (Dong et al., 2005; Yazar et al., 2020). Moreover, A151 can significantly reduce the levels of two key inflammatory factors, monocyte chemotactic protein 1 (MCP-1) and vascular cell adhesion molecule 1 (VCAM-1), in the inflammatory process of atherosclerosis (Cheng et al., 2008). Similarly, iSup ODN IRS954 could reduce the level of serum nucleic acid specific autoantibody and the grade of proteinuria, relieve the symptoms of glomerulonephritis and increase the survival rate of SLE mice (Barrat et al., 2007). Besides the inhibition of SIRS, SAT05F can also effectively reduce the deposition of immune complexes and delay the onset of lupus nephritis in mice with chronic graft-versus-host disease (GVHD), and inhibit the activation of TLR7/9 pathway *in vitro* (Zhang et al., 2014). Rheumatoid arthritis (RA) is an autoimmune chronic inflammatory joint disease. It was found that the PS modified ODN1411 could competitively inhibit the signal transduction of TLR8 by interacting with TLR8, that is, it inhibited the production of inflammatory factors in RA model by inhibiting the phosphorylation of signal molecules and the activation of NF-κB, which provided a possible new therapy for the treatment of RA (Sacre et al., 2016). In general, iSup-ODNs showed a good application prospect in IMIDs, especially the diseases associated with over-activation of immune response induced by TLR signal transduction (Li et al., 2022).

## 6 Conclusion

In the past two decades, a great deal of research has been done on the mechanism of inhibitory ODN in cells and its application in therapy, and great progress has been made in this field, providing potential therapeutic options for many medical problems that cannot be solved at present, and it is the most promising tool for gene targeted therapy at present. However, it is not difficult to find that there are still some problems in the research and development of inhibitory ODN drugs, such as easy to be degraded by ribozyme in the blood, and small molecular weight leads to fast renal clearance. Of course, appropriate modifications and efficient encapsulation are necessary for inhibitory ODNs to enhance the transport across membranes. In addition, off-target problem is also a non-negligible problem in drug development of inhibitory ODNs. Sometimes, inhibitory ODNs cannot accurately combine its corresponding target to achieve therapeutic effect due to the potential poor specificity and low affinity with the target. To solve the problem, it is necessary to modify inhibitory ODNs with longer length and more stable three-dimensional structure to enhance its binding specificity and affinity with the target.

Inhibitory ODN is a multi-faceted tool capable of delicate basic research and discovery that can later be molded into a therapeutic agent for clinical application. Reliability and reproducibility will be the pillars of future inhibitory ODN research as they are critical to successful clinical translation. With the increasing maturity of molecular genetics, pharmacology and chemical synthesis, as well as the rapid development of bioinformatics, we look forward to the discovery of more new mechanisms of action and the application of genomic information to ODN design, so further promote the application of inhibitory ODNs in the treatment of immune-mediated inflammatory diseases.
